# Inositol 1,4,5-Trisphosphate Signalling Regulates the Avoidance Response to Nose Touch in *Caenorhabditis elegans*

**DOI:** 10.1371/journal.pgen.1000636

**Published:** 2009-09-04

**Authors:** Denise S. Walker, Rafael P. Vázquez-Manrique, Nicholas J. D. Gower, Elizabeth Gregory, William R. Schafer, Howard A. Baylis

**Affiliations:** 1 Department of Zoology, University of Cambridge, Cambridge, United Kingdom; 2 MRC Laboratory of Molecular Biology, Cambridge, United Kingdom; Stanford University, United States of America

## Abstract

When *Caenorhabditis elegans* encounters an unfavourable stimulus at its anterior, it responds by initiating an avoidance response, namely reversal of locomotion. The amphid neurons, ASHL and ASHR, are polymodal in function, with roles in the avoidance responses to high osmolarity, nose touch, and both volatile and non-volatile repellents. The mechanisms that underlie the ability of the ASH neurons to respond to such a wide range of stimuli are still unclear. We demonstrate that the inositol 1,4,5-trisphosphate receptor (IP_3_R), encoded by *itr-1*, functions in the reversal responses to nose touch and benzaldehyde, but not in other known ASH-mediated responses. We show that phospholipase Cβ (EGL-8) and phospholipase Cγ (PLC-3), which catalyse the production of IP_3_, both function upstream of ITR-1 in the response to nose touch. We use neuron-specific gene rescue and neuron-specific disruption of protein function to show that the site of ITR-1 function is the ASH neurons. By rescuing *plc-3* and *egl-8* in a neuron-specific manner, we show that both are acting in ASH. Imaging of nose touch–induced Ca^2+^ transients in ASH confirms these conclusions. In contrast, the response to benzaldehyde is independent of PLC function. Thus, we have identified distinct roles for the IP_3_R in two specific responses mediated by ASH.

## Author Summary

In order to avoid potential hazards, animals detect and discriminate between a wide range of aversive stimuli. To detect some of these stimuli, animals use polymodal sensory neurons, that is neurons of a single type that can detect a range of different stimuli and transmit an appropriate signal to the downstream nervous system. Pain-sensing nociceptors in humans and the ASH neurons in *C. elegans* are both polymodal. The ASH neurons mediate responses to high osmotic strength, nose touch, high ambient oxygen, and volatile and non-volatile compounds. It remains unclear how these cells detect and discriminate between these different stimuli. We show that signalling through the second messenger inositol 1,4,5-trisphosphate (IP_3_) and its receptor (IP_3_R) is required in ASH for animals to respond to nose touch. We also show that IP_3_Rs are required for the response to the volatile compound benzaldehyde. However, these signalling components are not required for a range of other ASH-mediated responses. Thus, we have identified a signalling mechanism that is specific to a small subset of ASH-mediated responses. These results add to our understanding of how ASH discriminates between a variety of stimuli and thus to our understanding of polymodal neurons in general.

## Introduction

Like other animals, *C. elegans* negotiates its environment by responding to a range of noxious stimuli, by changing its direction of movement to avoid the source of the stimulus and thus avoid imminent injury. Mechanical stimulation is one type of stimulation that exerts such an effect. Depending on the position and strength of the mechanical stimulus, the neuronal circuitry responsible for this response differs. The response to nose touch relies primarily on the ASH pair of sensory neurons [1], which output to the command interneurons, AVA, AVB, AVD, AVE and PVC, which control forwards and backwards movement [2],[3]. These command neurons interact synaptically with one another and ultimately output to the motor neurons that control the body wall contractions necessary for sinusoidal movement. In contrast, the response to light anterior body touch relies on the ALM and AVM sensory neurons, which act upon these same command neurons.

The ASH neurons are particularly interesting in that they are polymodal nociceptive neurons, implicated in avoidance responses to a diverse range of sensory cues, namely, high osmotic strength, nose touch, high ambient oxygen, volatile compounds and non-volatile repellents such as heavy metals, protons and detergents [4]–[8]. ASH is thus analogous to human nociceptors, capable of responding to heat, mechanical stimulation and chemicals such as capsaicin. So understanding the signalling pathways that underlie the polymodal function of ASH is proving important to our understanding of human pain sensation. The molecular mechanisms that enable ASH to sense such a wide range of inputs are still poorly described. Work thus far has identified “general” components that are required for responses to all stimuli, and has also identified “specific” molecules that are required for single, or a small subset of, responses. The transient receptor potential vanilloid (TRPV)-related channel proteins OCR-2 and OSM-9 [9],[10], for example, appear to be required for all ASH-mediated responses, while GPA-3, a G-protein α subunit, is required for only a small subset [11]. Thus ASH utilises specific signalling pathways for individual stimuli, but these may converge on a common pathway.

**Figure 1:**
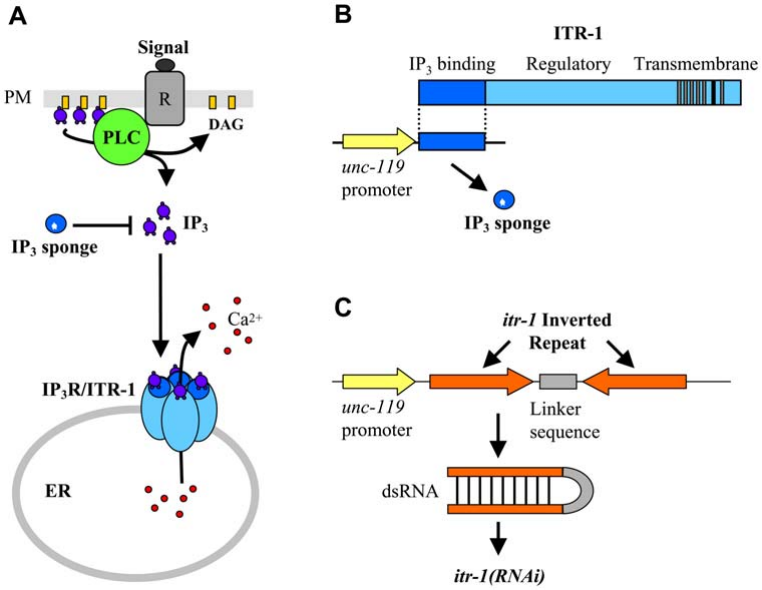
Disruption of IP_3_ signalling in the nervous system. (A) Schematic diagram showing the IP_3_ signalling cassette. Stimulation of a receptor (R) at the cell surface leads to the activation of phopholipase C (PLC), which catalyses the hydrolysis of phosphatidylinositol 4,5-bisphosphate to produce inositol 1,4,5-trisphosphate (IP_3_) and diacylglycerol (DAG). IP_3_ diffuses to the endoplasmic reticulum (ER), where it activates the IP_3_ receptor (IP_3_R), resulting in the release of Ca^2+^ into the cytoplasm. Expression of an IP_3_ sponge [see (B)] should mop up free IP_3_, thus interfering with its ability to activate IP_3_Rs. (B) Strategy used to disrupt IP_3_ signalling in the nervous system using IP_3_ sponges. ITR-1, the *C. elegans* IP_3_R subunit, consists of 3 functional regions, including an IP_3_ binding domain. Overexpression of the binding domain allows it to act as an IP_3_ sponge. Expression under the control of the *unc-119* promoter leads to nervous system-wide expression of the IP_3_ sponge. (C) Strategy used to express dsRNA, and thus disrupt *itr-1* expression, in the nervous system. Forward and reverse copies of an *itr-1* cDNA fragment are expressed under the control of the *unc-119* promoter. A “linker” region allows the complementary RNA regions to form dsRNA.

In the present study, we identify signalling through the inositol 1,4,5-trisphosphate receptor (IP_3_R) (Figure 1A) as a specific component, required for a small subset of ASH-mediated responses. IP_3_Rs in *Caenorhabditis elegans* are encoded by a single gene, *itr-1*, and are widely expressed throughout the animal, including in the nervous system [12]–[14]. A wide range of functions for *itr-1* have been identified. Genetic approaches have identified roles for *itr-1* in ovulation and meiotic maturation ([13],[15],[16], defecation [13],[16],[17], male mating [18] and in ventral enclosure [19]. We used a dominant-negative construct (IP_3_ sponge), as well as loss-of-function mutants and RNA interference, to demonstrate that IP_3_ signalling and IP_3_Rs function in the regulation of pharyngeal pumping rate and in multiple stages of embryogenesis [20].

For some of these functions, we have some insights into the nature of events upstream of *itr-*1 and in particular into the member(s) of the phospholipase C (PLC) family responsible for IP_3_ production (Figure 1A). For example, PLC-3 (PLCγ) appears to act upstream of ITR-1 in the regulation of gonadal sheath contraction (with the receptor tyrosine kinases LET-23 and VAB-1 presumably acting further upstream, [16]), and also during the defecation motor program [17]. We also know that PLC-1 (PLCε) acts upstream of ITR-1 to regulate ventral enclosure [21]. Finally, there is evidence that EGL-8 (PLCβ) functions upstream of ITR-1 in the control of sperm transfer [18].

In the present study we have used transgenic approaches to disrupt either IP_3_ signalling or *itr-1* function in the nervous system, and demonstrated a role in the avoidance responses to nose touch and benzaldehyde. Our evidence indicates that, for nose touch, two PLCs, PLC-3 and EGL-8, act as the source of IP_3_ upstream. We use cell-specific expression of an IP_3_ sponge and cell-specific rescue to show that *itr-1* and both PLCs are acting in the ASH neurons; and demonstrate that all three genes function in the production of nose touch-induced Ca^2+^ transients in ASH. Thus we have identified signalling components that are specific to two of the group of stimuli sensed by ASH.

## Methods

### Strains and constructs

The *C. elegans* strains used in this study are listed in Table S1. Strains containing the *plc-3(tm1340)* allele were maintained as balanced heterozygous strains, and assayed as homozygotes. *itr-1, egl-8* and *plc-3* strains carrying the *sra-6*p::YC2.12 construct were made by crossing the appropriate strain with AQ1444 [22]. Strains carrying *itr-1*(*sy290*gf) also carry a closely linked allele of *unc-24*, *unc-24(e138)*, which results in a locomotion (weak kinker) phenotype, which may interfere with the avoidance response. When using this allele we therefore rescued the *unc-24* deficiency by transgenic expression of a genomic fragment containing the wild type *unc-24* gene under the control of its own promoter. As a control, we rescued *unc-24(e138)* animals in the same way [18].

To construct *sra-6*, *glr-1* and *unc-119* promoter plasmids, we used 3.8 Kb [23], 5.2 Kb [24] and 1.3 Kb [25], respectively, of upstream DNA. IP_3_ sponge derivatives were constructed as described previously [20]. RNAi inverted repeat constructs were constructed using pHAB200 [12] by inserting forward and reverse copies of the same region of *E. coli lacZ* or *itr-1* cDNA either side of a “linker” made from *gfp* or a unique part of *lacZ*, respectively. *plc-3* rescue plasmids were constructed using a full length genomic fragment. *itr-1* and *egl-8* rescue plasmids were constructed using the Gateway system (Invitrogen), using the full-length cDNA (*itr-1*) or a “minigene (*egl-8*, as in [26]). These were introduced, along with the relevant promoter, into the destination vector pHP2 (see Table S1).

Constructs were introduced into *C. elegans* by injection [27] with a *mec-7p::gfp* marker plasmid, pPD117.01 (a gift from A. Fire).

### RNA-mediated interference (RNAi) of *itr-1*

RNA-mediated interference (RNAi) of *itr-1* was carried out using *E. coli* HT115 carrying derivatives of the vector pPD129.36 [28], which contains two flanking T7 RNA polymerase promoters. For RNAi of PLC genes we used derivatives of pPD129.36, pHAB301 (*egl-8*) and pHAB303 (*plc-3*) [18]. As a control we used a derivative of pPD129.36 with an *E. coli* chloramphenicol acetyltransferase (CAT) DNA insert. Plasmids were transformed into *E. coli* HT115 (DE3) and these strains used to perform RNAi feeding experiments [28].

### Behavioural assays

The response to nose touch was assayed on food at 20°C using an eyelash, as described by Kaplan and Horvitz [1]. The response to anterior body touch was assayed similarly. Reversal responses (initiated within 3 seconds of the stimulus) were quantified as distance reversed, expressed in worm lengths. Three categories of response were used, >1 worm length, which corresponds to a “good” reversal response, >0.1 worm length and 0 worm lengths, considered “poor” avoidance responses. A minimum of 40 animals were assayed for each genotype or condition shown. The response to repellents was assayed using the “dry drop” test [6], except for octanol, which was assayed using a “smell-on-a-stick” assay [23], as described by Chao *et al.* [29]. Results were analysed using Chi-squared tests.

### *In vivo* Ca^2+^ imaging

Optical recordings were performed essentially as described [30],[31] on a Zeiss Axioskop 2 upright compound microscope equipped with a Dual View beam splitter and a Uniblitz Shutter. The following filters and dichroics were used: excitation: 400–440 nm bandpass; excitation dichroic: 455 nm; CFP emission: 465–495 nm bandpass; emission dichroic: 505 nm; YFP emission: 520–550 nm bandpass. Individual adult worms (∼24 h past L4) were glued with Nexaband S/C cyanoacrylate glue to pads composed of 2% agarose in extracellular saline (145 mM NaCl, 5 mM KCl, 1 mM CaCl_2_, 5 mM MgCl_2_, 20 mM D-glucose, 10 mM HEPES buffer, pH 7.2, 2 mM serotonin). Fluorescence images were acquired using MetaVue 6.2. Acquisitions were taken at 28 Hz (35 ms exposure time) with 4×4 or 2×2 binning, using a 63× Zeiss Achroplan water immersion objective.

Nose touch stimulation was performed as described [32]. A rounded glass needle was placed perpendicular to the worm's body at a distance of 150 µm from the side of the nose, displaced 8 µm into the side of the worm's nose, held in position for 1 second, and then pulled back to its original position. For each strain, we recorded 2 responses for 10 animals, with 5 minutes between stimuli. Results were compared using a Mann-Whitney rank sum test.

## Results

### Disruption of *itr-1* function in the nervous system

In order to investigate the role of IP_3_ signalling in the nervous system, we expressed the cDNA encoding the IP_3_ binding domain of *itr-1* (an “IP_3_ sponge”, [20]) under the control of the promoter of *unc-119*, which is widely, and exclusively, expressed in the nervous system [25]. Two derivatives of the IP_3_ sponge were used, as described previously [33]. The “control sponge” (K579Q, R582Q), is deficient in IP_3_ binding and therefore should not disrupt IP_3_ signalling, while the “super sponge” (R511C) has increased affinity for IP_3_. In a second approach, to disrupt IP_3_R function rather than IP_3_ signalling, we used the *unc-119* promoter to control expression of an *itr-1* dsRNAi “snapback” construct [34]. Figure 1 illustrates these approaches.

### IP_3_ signalling and *itr-1* function in the aversive response to nose touch

We determined the role of IP_3_ signalling and *itr-1* in the avoidance response to nose touch. Mechanical stimuli were delivered to the nose of moving animals using an eyelash, essentially as described by Kaplan and Horvitz [1]. In order to detect differences in the type of movement response exhibited, we used a scoring system in which the length of reversal was expressed in worm lengths (see Methods). Three categories of response were used, >1 worm length, >0.1 worm length and 0 worm lengths. The first is considered a “good” response, while the latter two correspond to “poor” responses. This scoring method is similar to that used by Kindt *et al*. [32], with the >0.1 worm length category usually corresponding to a “head withdrawal” response [32],[35].

**Figure 2:**
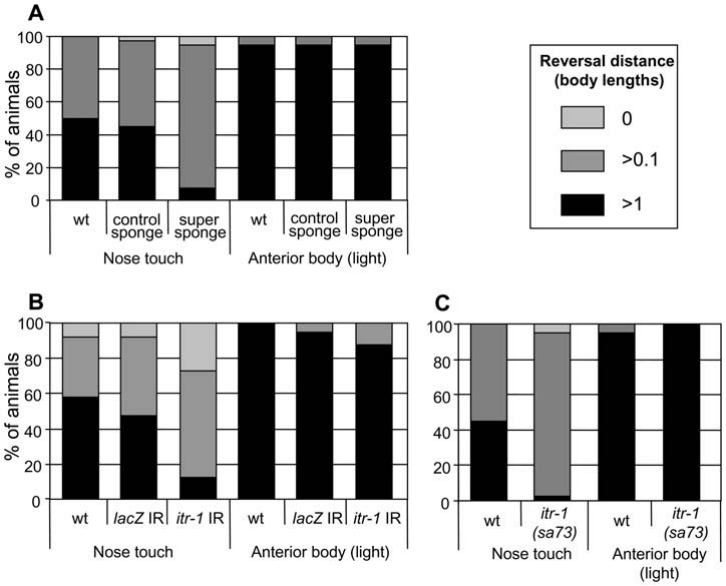
IP_3_ and *itr-1* function in the aversive response to nose touch. The reversal response (measured in worm lengths) of animals exposed to nose touch or light anterior body touch. (A) Animals expressing IP_3_ sponge derivatives under the control of the *unc-119* promoter. (B) Animals expressing dsRNA under the control of the *unc-119* promoter. IR, inverted repeat. (C) Animals carrying the *itr-1(sa73)* loss-of-function allele. All three methods of disrupting ITR-1 function significantly disrupt the nose touch response in comparison to wt animals (P<0.001, Chi-squared test, in each case). The control sponge (A) and *lacZ* control IR (B) do not disrupt the response (P>0.05).

As Figure 2A shows, when cDNA encoding the IP_3_ super sponge is expressed under the control of the *unc-119* promoter, the reversal response to nose touch is severely disrupted, while expression of the control sponge in the same way has no effect. However, the response to light anterior body touch, which uses a different neuronal circuitry but relies on the same command neurons and muscle groups, remains unaffected, indicating that the defect is specific to nose touch, rather than a general movement defect. The avoidance responses to harsh anterior body touch and both harsh and light posterior body touch are similarly unaffected (DSW and HAB, unpublished). Thus, IP_3_ signalling functions in the avoidance response to nose touch.

As Figure 2B shows, when an *itr-1* dsRNAi “snapback” construct is expressed in the nervous system, the response to nose touch is significantly disrupted. A dsRNAi construct for the *E. coli lacZ* gene, expressed in the same way, has no effect. As Figure 2C shows, *itr-1(sa73)* (temperature sensitive, loss-of-function) animals also demonstrate a defective response to nose touch at 20°C, a partially restrictive temperature. In both cases, the response to light anterior body touch is unaffected, as were the responses to other types of mechanical stimuli (DSW and HAB, unpublished). Since the vast majority of animals still exhibit a slight movement response (>0.1 worm length), head withdrawal appears to be unaffected. Thus, *itr-1* functions specifically in the reversal response to nose touch.

### *itr-1* functions in the avoidance response to a volatile repellent, benzaldehyde, but not in other ASH-mediated responses

**Figure 3:**
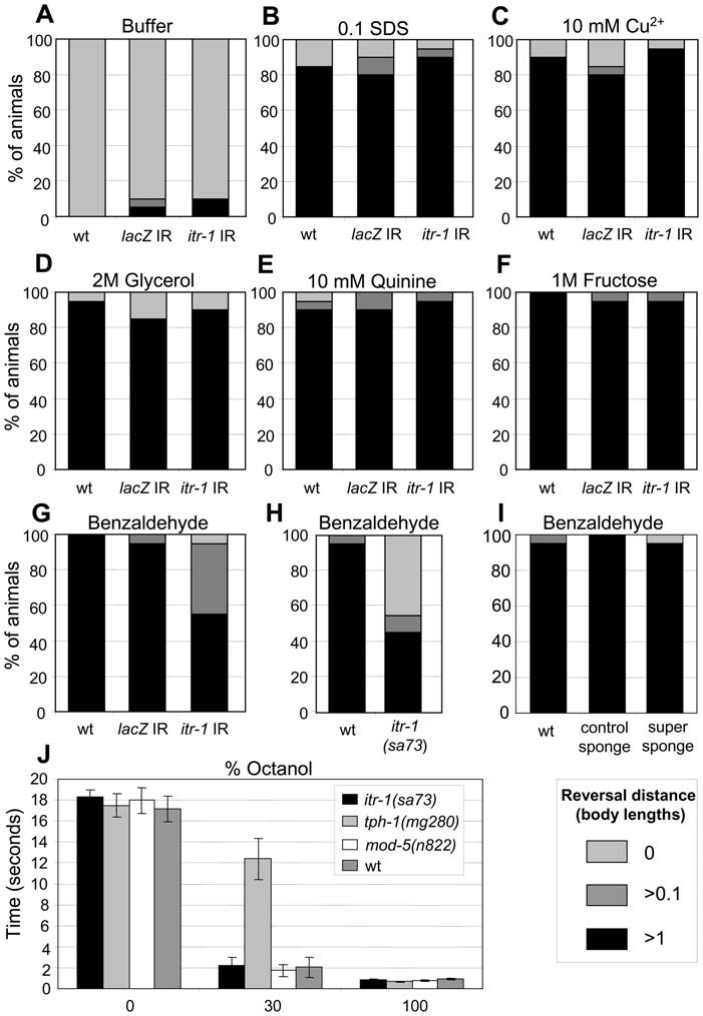
*itr-1* functions in the avoidance response to a volatile repellent, benzaldehyde, but not in other ASH-mediated responses. (A–I) The reversal response of animals (measured in worm lengths; see key, bottom right) exposed to a range of stimuli. (A–F) Reversal responses in animals expressing *itr-1* or *lacZ* dsRNA (IR, inverted repeat) under the control of the *unc-119* promoter and treated with: (A) Buffer alone (30 mM Tris [pH 7.5], 100 mM NaCl, 10 mM KCl), (B) SDS, (C) copper, (D) glycerol, (E) quinine, and (F) fructose, at the concentrations indicated, using a “dry drop” assay [6]. (G–I) Reversal response to benzaldehyde (undiluted) of (G) animals expressing dsRNA under the control of the *unc-119* promoter (IR, inverted repeat); (H) wild type and *itr-1* loss-of-function animals; (I) animals expressing IP_3_ sponge derivatives under the control of the *unc-119* promoter. (J) Response to octanol, measured as the time taken to reverse, following administration of the octanol concentrations indicated, as a “smell-on-a-stick” [29]. Animals in which *itr-1* is knocked down in the nervous system, or which carry the *itr-1(sa73)* mutation are defective in the response to benzaldehyde [P<0.001, (G)] but not to other repellants (P>0.05). However, the IP_3_ sponge failed to disrupt the response to benzaldehyde [P>0.05, (I)]. All P values are from Chi-squared tests.

The response to nose touch is largely mediated through the ASHL and ASHR pair of amphid sensory neurons, although minor roles appear to be played by FLP and OLQ neurons [1]. The ASH neurons are polymodal in function, with roles identified not only in the avoidance of nose touch, but also in the avoidance of high osmolarity and both volatile and non-volatile repellents [4],[6],[8]. To determine whether *itr-1* has a global role in ASH responses or is specifically required for nose touch, we tested whether it has a similarly important role in other responses known to be mediated by ASH. As Figure 3A–3F shows, expression of the *itr-1* dsRNAi construct under the control of the *unc-119* promoter does not significantly disrupt the avoidance responses to high osmolarity (fructose), SDS, copper, quinine or glycerol (although our experiments do not exclude more subtle roles). However, the response to the volatile repellent benzaldehyde is disrupted (Figure 3G). Similarly, *itr-1(sa73)* animals display a defective response to benzaldehyde (Figure 3H). Interestingly, however, the use of an IP_3_ sponge failed to disrupt the aversive response to benzaldehyde (Figure 3I), suggesting that this response could be independent of IP_3_. We tested another volatile repellent, octanol, and found that, in the presence of food, the responses to 30% and 100% octanol are unaffected (Figure 3J) whilst wild type, and *tph-1* and *mod-5* mutants, behave as expected [29]. Thus *itr-1* appears to function in a very limited subset of ASH-mediated avoidance responses, to nose touch and benzaldehyde.

### PLCβ and PLCγ function in the aversive response to nose touch through the production of an IP_3_ signal

**Figure 4:**
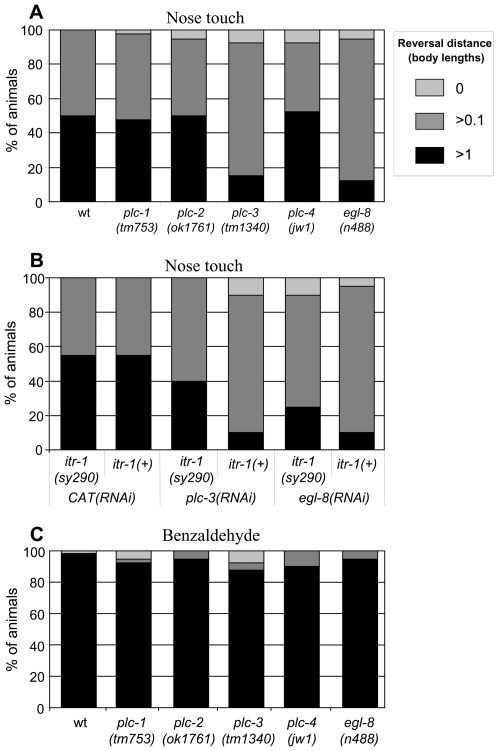
PLCβ and PLCγ function, through the production of an IP_3_ signal, in the aversive response to nose touch, but not to benzaldehyde. (A) Reversal response of PLC deficient animals to nose touch. All showed a wild type response (>90% reversing >1 worm length) to anterior body touch (data not shown). (B) Reversal response of *itr-1(sy290)* gain-of-function animals to nose touch, following depletion of *plc-3* or *egl-8* by RNAi. CAT, *E. coli* chloramphenicol acetyltransferase. All showed a wild type response (>90% reversing >1 worm length) to anterior body touch (data not shown). (C) Reversal response of PLC deficient animals to benzaldehyde. All showed a wild type response (<5% reversing >1 worm length) to buffer alone (data not shown). Both *plc-3* and *egl-8* loss-of-function mutants exhibit defective responses to nose touch (P<0.001) compared to wt animals. RNAi of *plc-3* or *egl-8* similarly disrupted the response (P<0.001) compared to the *CAT(RNAi)* control animals. However, RNAi of *plc-3* or *egl-8* in an *itr-1(sy290)* background failed to disrupt the response to such an extent (*plc-3*, P<0.001; *egl-8*, P<0.05, when compared to RNAi of the same genes in wt animals). All P values are from Chi-squared tests.

PLCs catalyse the hydrolysis of PIP_2_, to produce IP_3_ (Figure 1) and are therefore good candidates for the source of signal that activates the IP_3_R. We therefore investigated the role of *C. elegans* PLCs in the avoidance response to nose touch. In *C. elegans* five PLC genes and one further PLC-like gene have been identified in the genome [18]. They correspond to vertebrate PLC-β (*egl-8* [26],[36],[37]), PLC-δ, PLC-γ (*plc-4* and *plc-3*, respectively [16]), PLC-ε (*plc-1* [37]), and an unusual, β-like protein (*plc-2* [18]). As Figure 4A shows, both *egl-8* and *plc-3* loss-of-function mutants exhibit a significant defect in the aversive response to nose touch, while loss-of-function mutants for the other PLC genes remain unaffected. The response to light anterior body touch is unaffected, as were the responses to other types of stimuli (data not shown). Thus, *egl-8* and *plc-3* function specifically in the aversive response to nose touch.

Since PLC-β and PLC-γ catalyse the production of two second messengers, IP_3_ and diacylglycerol (DAG), we wished to demonstrate that it is via the generation of an IP_3_ signal that they function in the nose touch response. To this end, we investigated whether *itr-1(sy290)*, a gain-of-function allele [38], could rescue the defects in nose touch response that resulted from *egl-8* or *plc-3* RNAi. *itr-1(sy290)* has a mutation, R582Q, in the IP_3_ binding site [38], which results in a two-fold increase in IP_3_ binding affinity [20]. As Figure 4B shows, RNAi of *plc-3* and of *egl-8* (in a wild type *itr-1* background) is able to reproduce the defect in nose touch response that was observed for loss-of-function mutants. However, RNAi of *egl-8* and *plc-3* on *itr-1(sy290)* animals failed, significantly, to disrupt the nose touch response to such an extent. Thus, an *itr-1* mutation that increases the receptor's affinity for IP_3_ partially rescues the defects in nose touch response that result from knockdown of either *plc-3* or *egl-8*, suggesting that IP_3_ is an important component of the downstream signal from these PLCs.

We investigated the role of PLCs in the response to benzaldehyde. As Figure 4C shows, the response to benzaldehyde remained intact in all of the PLC loss-of-function mutants. As both *egl-8* and *plc-3* are implicated in the response to nose touch we also attempted to test a *plc-3*,*egl-8*, double mutant for responses to benzaldehyde, however the double mutant animals had severe locomotive defects and we were not able to perform the relevant assays. Thus it appears that the response to benzaldehyde, although IP_3_R-dependent, may be independent of PLC function. Although we cannot rule out that the action of PLCs is redundant in this response, this data is compatible with the suggestion that this response does not depend on IP_3_ (Figure 3I).

### *itr-1* functions in ASH

**Figure 5:**
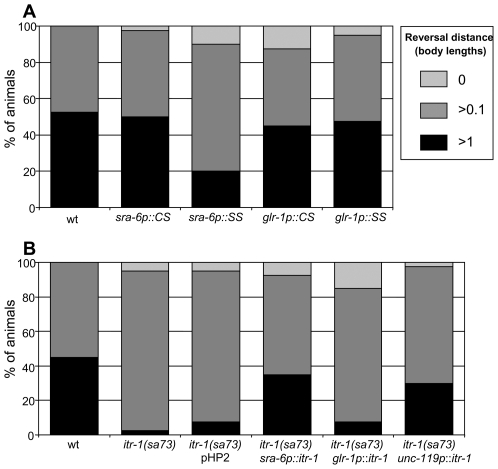
*itr-1* functions in ASH. Reversal response to nose touch. (A) Animals expressing IP_3_ sponge derivatives under the control of cell-specific promoters. CS, control sponge; SS, super sponge. (B) *itr-1(sa73)* loss-of-function animals expressing full-length *itr-1* cDNA under the control of cell-specific promoters. pHP2 is the empty destination vector used in construction of the other plasmids. All showed a wild type response (>90% reversing >1 worm length) to anterior body touch (data not shown). When the IP_3_ sponge is expressed under control of the *sra-6* promoter, nose touch response is disrupted (P>0.001), while expression under control of the *glr-1* promoter has no effect (P>0.05). Expression of the control sponge using either promoter has no effect (P>0.05). All P values are from Chi-squared tests.

The most likely candidates for the site of action of *itr-1* in the nose touch response are the ASH neurons themselves or the downstream command neurons. In order to distinguish between these possibilities (and the alternative, which is that it functions elsewhere to influence the function of these neurons in some way), we exploited the availability of neuron-specific promoters. The promoter of *sra-6* directs expression in ASH, and (weakly) in ASI and PVQ [23], while that of *glr-1* directs expression in the command neurons AVA, AVB, AVD, AVE and PVC and several others, but not in ASH [35]. We therefore used these promoters to carry out cell-specific rescue and disruption of *itr-1* function. As Figure 5A shows, when the IP_3_ super sponge is expressed under control of the *sra-6* promoter, the response to nose touch is disrupted. In contrast, when it is expressed under control of the *glr-1* promoter the response is unaffected. Likewise, expression of the control sponge, using either promoter, does not disrupt the response. Thus, disruption of IP_3_ signalling in ASH, but not the command neurons, interferes with the response to nose touch.

We also used the opposite approach, expressing *itr-1* cDNA under control of specific promoters and assessing whether this could rescue the defect in nose touch response that is observed in JT73 animals, which carry the *itr-1(sa73)* loss-of-function mutation. As Figure 5B shows, expression of *itr-1* under the control of the *sra-6* promoter rescues the defect seen in *itr-1(sa73)* animals, as does the expression of *itr-1* under control of the pan-neuronal *unc-119* promoter. In contrast, expression of *itr-1* using the *glr-1* promoter failed to rescue this defect. Thus, the defect in the reversal response to nose touch observed in *itr-1(sa73)* animals can be rescued by expression of *itr-1* cDNA in ASH, but not in the command neurons.

### *plc-3* and *egl-8* function in ASH

**Figure 6:**
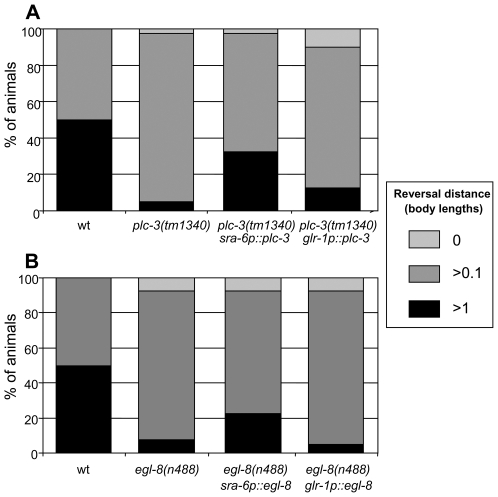
*egl-8* and *plc-3* function in ASH. Reversal response to nose touch. (A) *plc-3(tm1340)* loss-of-function animals expressing *plc-3* genomic DNA under the control of cell-specific promoters. (B) *egl-8(n488)* loss-of-function animals expressing an *egl-8* rescuing “minigene” under the control of cell-specific promoters. All showed a wild type response (>90% reversing >1 worm length) to anterior body touch (data not shown). Expression of *plc-3* or *egl-8* under control of the *sra-6* promoter significantly rescues nose touch response in their respective mutants (P<0.001), while expression using the *glr-1* promoter does not (P>0.05). All P values are from Chi-squared tests.

Since *plc-3* and *egl-8* appear to act upstream of *itr-1*, we hypothesised that they also effect their role in the nose touch response in the ASH neurons. To test this, we used *sra-6* and *glr-1* promoters to rescue *plc-3* and *egl-8* in a neuron-specific manner in loss-of-function mutants. As Figure 6A shows, when *plc-3* is expressed under the control of the *sra-6* promoter in *plc-3(tm1340)* homozygotes, the defect in nose touch response is significantly rescued, while expression of *plc-3* under the control of the *glr-1* promoter fails to rescue. Thus, as predicted, the site of *plc-3* function in the response to nose touch also appears to be the ASH neurons.

As Figure 6B shows, when *egl-8* is expressed under the control of the *sra-6* promoter in *egl-8(n488)* animals, the defect in nose touch response is significantly rescued, while expression of *egl-8* under the control of the *glr-1* promoter fails to rescue. Thus the site of *egl-8* function in the response to nose touch is also the ASH neurons.

### *itr-1*, *egl-8*, and *plc-3* function in nose touch–induced Ca^2+^ transients in ASH

**Figure 7:**
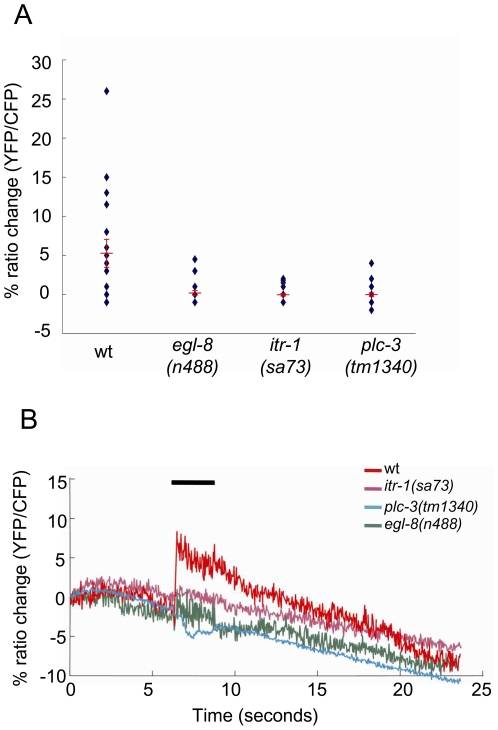
*itr-1*, *egl-8*, and *plc-3* all function in nose touch–induced Ca^2+^ transients in ASH. Ratio changes in cameleon-expressing ASH neurons, following nose touch. (A) Quantification of responses. Diamonds are individual observations; longer red lines are mean; error bars are s.e.m. (n = 20). (B) Representative responses, for wild type animals and the mutants indicated. Black bar indicates duration of stimulation. The CFP/YFP ratio decreases over the course of the recordings because YFP photobleaches faster than CFP; noise is relatively low in some animals, due to higher cameleon expression levels. Nose touch–evoked Ca^2+^ transients were significantly disrupted in *itr-1*, *egl-8*, and *plc-3* loss-of-function animals (P<0.05, Mann-Whitney rank sum test).

In order to more directly observe how *itr-1*, *egl-8* and *plc-3* affect sensory responses in ASH, we used the genetically encoded Ca^2+^ sensor, cameleon, to examine *in vivo* calcium transients evoked by nose touch in ASH. As previously observed [22],[32], mechanical stimulation of the nose evoked calcium influx in the ASH neurons of wild-type animals expressing cameleon under control of the *sra-6* promoter (Figure 7). However, nose touch-evoked calcium transients were significantly disrupted in *itr-1* mutant animals, indicating that the IP_3_ receptor is required for ASH mechanosensory responses. Likewise, mutants defective in *egl-8* or *plc-3* showed significantly reduced calcium transients in ASH in response to nose touch. Together, these results indicate that the IP_3_ pathway is required for nose touch mechanosensation in ASH.

## Discussion

We have shown that signalling through IP_3_Rs is required for aversive responses to nose touch and benzaldehyde in *C. elegans*. The response to nose touch requires *itr-1* function and the action of *plc-3* and *egl-8* in the polymodal ASH neurons, where they function in the generation of Ca^2+^ transients. The ability of the IP_3_ sponge to disrupt nose touch and the rescue of nose touch defects in *plc-3* and *egl-8* RNAi animals by an *itr-1* gain-of-function allele, both support the conclusion that IP_3_ is the signalling molecule downstream of PLC and upstream of *itr-1* activation. Thus nose touch requires a canonical IP_3_ signalling pathway in ASH.

ASH neurons display striking polymodality and clearly distinguish functionally between different stimuli. For example, habituation to repeated nose touch has no effect on the response to octanol or high osmotic strength [39]. Likewise, prolonged exposure to copper affected behavioural and neural responses to copper but not to other repellents detected by ASH such as glycerol [22]. The ability of ASH neurons to discriminate between different aversive stimuli and undergo stimulus-specific adaptation indicates that at some level ASH uses different sensory transduction mechanisms for different modalities. At the molecular level, genes which are required for subsets of responses have been identified (see review in [40]). For example, OSM-10 is required to sense osmolarity but not for other sensory responses [39], while GPA-3 specifically affects acute responses to quinine [22]. Our new results identify the IP_3_ pathway as playing a specific role in the mechanosensory modality of ASH.

Interestingly, *itr-1* also affects a second ASH-dependent behaviour - avoidance of high concentrations of benzaldehyde. Thus it would be interesting to determine to what extent these responses show segregation or interact, for example whether habituation to nose touch alters responses to benzaldehyde or *vice versa*. The molecular overlap between the responses to these two very different stimuli is intriguing. Although both require *itr-1* they do show some differences. The response to benzaldehyde is not disrupted by the use of IP_3_ sponges, and appears not to be dependant on PLC (although we cannot eliminate the possibility that *egl-8* and *plc-3* act redundantly). This would suggest that, although both responses use the IP_3_R, the upstream components may be different. One explanation for these results is that the benzaldehyde response is mediated by an IP_3_-independent mechanism. IP_3_ independent activation of IP_3_Rs by proteins ligands such as CaBP (Ca^2+^ binding protein), CIB1 (Ca^2+^ and integrin binding 1, also known as calmyrin) and G-protein βγ subunits has been shown in other systems [41], Homologues of CIB1 and Gβγ are both present in worms so such mechanisms could act within ASH. It would be interesting to know whether the site of *itr-1* function in the benzaldehyde response is also ASH. However, due to technical limitations, we have been unable to test this. The profound movement defects of *egl-30* mutants have also prevented us from testing whether this response is also Gαq-independent.

The TRPV channels OSM-9 and OCR-2 are required for all ASH mediated responses, suggesting that response-specific signalling pathways converge on a common mechanism of activation. However, how the detection of such a wide range of stimuli is coupled to gating of these channels is unclear. The identification of signalling components that are required for detection of single stimuli, or small subsets, is vital to resolving this issue. OSM-10, for example, is only required for detection of osmotic stimuli [39]. Similarly we have shown that *itr-1* is only required for two responses, nose touch and benzaldehyde. It remains to be established how *itr-1* mediated signals are coupled to the activation of OSM-9 and OCR-2. It is probable that signals downstream of *itr-1* are transduced by Ca^2^+ released from the ER. Many TRP channels are regulated by calmodulin, a key target of intracellular Ca^2+^ release. Calmodulin can act as both a positive and negative regulator of TRP channels [42]. Interestingly, TRPV4 and 6 are both positively regulated by CaM. The TRPV4 C-terminal CaM binding site which is required for positive regulation [43] shows some conservation with OSM-9 (HAB, unpublished). A range of other signals are known to be involved in regulating TRP channel function. For example polyunsaturated fatty acids (PUFAs) are known to play an important role in regulating the activity of some TRP channels [44] and it has been suggested that PUFAs play a key role in regulating OSM-9/OCR-2 function [45]. Thus *itr-1* might also regulate OSM-9/OCR-2 indirectly through other pathways.

Our results place *egl-8* (PLC-β) and *plc-3* (PLC-γ) as being upstream of *itr-1*. In each case we observed partial rescue when the genes were expressed in ASH in loss-of-function backgrounds. This partial rescue may be due to inadequate expression from the *sra-6* promoter or could reflect a requirement for these genes in other cells, although we do not observe any rescue on expression in command neurons. The identification of a role for two PLC subtypes is at first glance surprising, however there are many examples of multiple PLC subtypes being utilised in physiological processes (see for example [17]). In ASH our results suggest that both act, at least in part, through IP_3_. The signals upstream of PLC are unknown. PLC-β is usually regulated by members of the Gqα subunits of heterotrimeric G-proteins. We were unable to test the role of EGL-30 (Gqα) in this process as egl*-30* mutants have widespread defects in locomotion. In addition, ASH expresses at least 9 G-alpha subunits. Some of these have identified and specific functions whilst some are more general; e.g. *odr-3* appears to be required for all known ASH-mediated responses, while *gpa-3* is only required for the response to water soluble repellents [11],[46]. Whether any of the other Gα subunits are specifically required in nose touch remains unclear. We tested the effect of mutations in the Gα subunits expressed in ASH, however, we found that most impaired nose touch, to varying degrees (DSW and HAB, unpublished data). It seems likely that their role is complex, involving multiple cells types and perhaps redundancy between subunit types.

How does PLC and IP_3_ signalling facilitate the response to nose touch? The mechanism by which ASH neurons detect nose touch is unclear. Kindt *et al.* [32] have shown that the response of two other neurons QLQ and Il1 to nose touch involves the mechanosensitive TRPA channel TRPA-1. However TRPA1 does not appear to be required in ASH [32]. Thus mechanosensation in ASH may use a different mechanism. One possibility is that IP_3_ signalling in ASH lies downstream of ligand independent activation of GPCRs. Analysis of the “Bayliss Response” in which small resistance arterial blood vessels constrict in response to rises in blood pressure has identified a pathway which is initiated by the activation of GPCRs by membrane stretch [47]. In these vascular smooth muscle cells ligand independent activation of the Angiotensisn II AT1 receptor by membrane stretch regulates a TRP channel, TRPC6, through a mechanism that requires both G_αq_ and PLC. Other G_αq_ linked GPCRs also demonstrate mechanosensitive properties [47]. The signal between PLC and TRPC6 has not been identified. As discussed above we have shown that in ASH, nose touch is mediated by PLC-β (*egl-8*) which is normally downstream of G_αq_ coupled GPCRs so our data are compatible with such a mechanism operating in these cells. Alternatively, IP_3_ signalling might not be directly activated by nose touch. Many mechanosensory processes involve ion channels that are directly activated by force; thus, IP_3_ signalling might regulate the activity of a mechanosensitive channel responsible for sensing nose touch in ASH. In this model, IP_3_ signalling does not mediate sensory transduction *per se*, but rather acts downstream of G-protein-mediated neuromodulatory pathways to modify touch sensitivity. Pathways of this sort would be critical for modality-specific adaptation in a polymodal neuron such as ASH.

In summary, we have shown that the IP_3_ signalling cassette is part of the specific signalling machinery for nose touch in ASH neurons. This adds to our molecular understanding of the molecular mechanisms that enable the segregation of signals in these polymodal sensory neurons and contributes to our understanding of how polymodal neurons, such as human nociceptors, function in general.

## Supporting Information

Table S1 Strains used in this work.
(0.09 MB DOC)
