# Supplementary material for: Inositol 1,4,5-Trisphosphate Signalling Regulates the Avoidance Response to Nose Touch in Caenorhabditis elegans
Source: PLoS Genet. 2009 Sep 4;5(9):e1000636. doi: 10.1371/journal.pgen.1000636 (PMC2729924; doi:10.1371/journal.pgen.1000636)
Supplement: Table S1 — Strains used in this work. (0.09 MB DOC) [file pgen.1000636.s001.doc]

| **Table S1.** **Strains used in this work** | | |
| --- | --- | --- |
| **Strain^a^** | **Genotype** | **Reference^b^** |
| N2 | wild type | [1] |
| CB138 | *unc-24(e138)IV* | [1] |
| GR1321 | *tph-1(mg280)II* | [2] |
| HB101 | *itr-1(sy290) unc-24 (e138)IV*; *him-5* (*e1467*)*V*; *jwEx101 [unc-24 (+), mec-7::gfp]* | [3] |
| HB103 | *unc-24(e138)IV*; *him-5* (*e1467*)*V*; *jwEx101 [unc-24 (+), mec-7::gfp]* | [3] |
| HB119 | *plc-3(tm1340)/mIn1[plc-3(+) mIs14]II; jwEx119 [sra-6p::plc-3(+)::yfp, mec-7p::gfp]* | This work |
| HB120 | *plc-3(tm1340)/mIn1[plc-3(+) mIs14]II; jwEx120 [glr-1p::plc-3(+)::yfp, mec-7p::gfp]* | This work |
| HB123 | *itr-1(sa73)IV ; jwEx123 [unc-119p::itr-1(+),mec-7p::gfp]* | This work |
| HB124 | *itr-1(sa73)IV ;*  *jwEx124* [pHP2 (Gateway destination vector)*, mec-7p::gfp]* ^c^ | This work |
| HB125 | *itr-1(sa73)IV ; jwEx125 [glr-1p::itr-1(+),mec-7p::gfp]* | This work |
| HB126 | *itr-1(sa73)IV ; jwEx126 [sra-6p::itr-1(+),mec-7p::gfp]* | This work |
| HB163 | *egl-8(n488)V ; jwEx163 [sra-6p::egl-8(+)::gfp, mec-7p::gfp]* | This work |
| HB164 | *egl-8(n488)V ;*  *jwEx164 [glr-1p::egl-8(+)::gfp, mec-7p::gfp]* | This work |
| HB169 | *jwEx169 [glr-1p::itr-1_1-2115_* R511C ( “super sponge”), *mec-7p::gfp* | This work |
| HB171 | *jwEx171 [sra-6p:: itr-1_1-2115_* R511C ( “super sponge”), *mec-7p::gfp]* | This work |
| HB173 | *jwEx173 [glr-1p:: itr-1_1-2115_* K579Q R582Q ( “control sponge”), *mec-7p::gfp]* | This work |
| HB175 | *jwEx175 [sra-6p:: itr-1_1-2115_* K579Q R582Q ( “control sponge”), *mec-7p::gfp]* | This work |
| HB176 | *jwEx176 [unc-119p::itr-1_1-2115_* R511C ( “super sponge”), *mec-7p::gfp]* | This work |
| HB177 | *jwEx177 [unc-119p::itr-1_1-2115_* R511C ( “super sponge”), *mec-7p::gfp]* | This work |
| HB178 | *jwEx178 [unc-119p:: itr-1*_187-1107_::*gfp*::*itr-1*_1107-187_ (inverted repeat), *mec-7p::gfp]* | This work |
| HB179 | *jwEx179 [unc-119p::lacZ*_1878-524_:: *lacZ* _1318-1948_ (inverted repeat), *mec-7p::gfp]* | This work |
| HB349 | *plc-4(jw1)IV* | [4] |
| HB372 | *plc-1(tm753)X* | [4] |
| HB652 | *plc-3(tm1340)II*/*mIn1*[*dpy-10(e128)mIs14*]*II* | [4] |
| HB679 | *plc-2(ok1761)V* | [4] |
| JT73 | *itr-1(sa73)IV* | [5] |
| MT1083 | *egl-8(n488)V* | [6] |
| MT8944 | *mod-5(n822)V* | [7] |
| PS2582 | *itr-1(sy290)* *unc-24(e138)IV* | [8] |
| ^a^ N2, CB138, DR2078, JT73, MT1083, PS2582 were supplied by the *Caenorhabditis* Genetics Centre (University of Minnesota, MN).  ^b^ References  1. Brenner S (1974) The genetics of *Caenorhabditis elegans*. Genetics 77: 71-94.  2. Sze JY, Victor M, Loer C, Shi Y, Ruvkun G (2000) Food and metabolic signalling defects in a *Caenorhabditis elegans* serotonin-synthesis mutant. Nature 403: 560-564.  3. Gower NJ, Walker DS, Baylis HA (2005) Inositol 1,4,5-trisphosphate signaling regulates mating behavior in *Caenorhabditis elegans* males. Mol Biol Cell 16: 3978-3986.  4. Vazquez-Manrique RP, Nagy AI, Legg JC, Bales OA, Ly S, et al. (2008) Phospholipase C-ε regulates epidermal morphogenesis in *Caenorhabditis elegans*. PLoS Genet 4: e1000043.  5. Iwasaki K, Liu DW, Thomas JH (1995) Genes that control a temperature-compensated ultradian clock in *Caenorhabditis elegans*. Proc Natl Acad Sci U S A 92: 10317-10321.  6. Trent C, Tsuing N, Horvitz HR (1983) Egg-laying defective mutants of the nematode *Caenorhabditis elegans*. Genetics 104: 619-647.  7. Ranganathan R, Sawin ER, Trent C, Horvitz HR (2001) Mutations in the *Caenorhabditis elegans* serotonin reuptake transporter MOD-5 reveal serotonin-dependent and -independent activities of fluoxetine. J Neurosci 21: 5871-5884.  8. Clandinin TR, DeModena JA, Sternberg PW (1998) Inositol trisphosphate mediates a RAS-independent response to LET-23 receptor tyrosine kinase activation in *C. elegans*. Cell 92: 523-533.  ^c^ pHP2 was produced from a Gateway destination vector (gift of Mario de Bono), which was formed originally by insertion of the Gateway cloning cassette and the *gcy-32* promoter into pPD49.26 (Andy Fire). To create pHP2, the *gcy-32* promoter was removed by digestion with *Xba*I and *Nhe*I followed by re-ligation of the vector. | | |
